# Advances in the diagnosis and treatment of *Clostridium difficile* infections

**DOI:** 10.1038/s41426-017-0019-4

**Published:** 2018-02-07

**Authors:** Zhong Peng, Lifen Ling, Charles W. Stratton, Chunhui Li, Christopher R. Polage, Bin Wu, Yi-Wei Tang

**Affiliations:** 1 State Key Laboratory of Agricultural Microbiology, College of Veterinary Medicine, Huazhong Agricultural University, Wuhan, 430070 Hubei China; 2 The Eighth Affiliated Hospital of Sun Yat-sen University, Shenzhen, 518000 Guangdong China; 3 Department of Laboratory Medicine, Memorial Sloan Kettering Cancer Center, New York, NY 10065 USA; 4 Department of Pathology, Microbiology and Immunology, Vanderbilt University Medical Center, Nashville, TN 37232 USA; 5 Infection Control Center, Xiangya Hospital of Central South University, Changsha, 410008 Hunan China; 6 Departments of Pathology and Laboratory Medicine and Internal Medicine, University of California Davis School of Medicine, Sacramento, CA 95817 USA; 7 Department of Pathology and Laboratory Medicine, Weill Medical College of Cornell University, New York, NY 10065 USA

## Abstract

*Clostridium difficile* is a leading cause of antibiotic-associated diarrhea worldwide. The diagnosis of *C. difficile* infection (CDI) requires both clinical manifestations and a positive laboratory test for *C. difficile* and/or its toxins. While antibiotic therapy is the treatment of choice for CDI, there are relatively few classes of effective antibiotics currently available. Therefore, the development of novel antibiotics and/or alternative treatment strategies for CDI has received a great deal of attention in recent years. A number of emerging agents such as cadazolid, surotomycin, ridinilazole, and bezlotoxumab have demonstrated activity against *C. difficile*; some of these have been approved for limited clinical use and some are in clinical trials. In addition, other approaches such as early and accurate diagnosis of CDI as well as disease prevention are important for clinical management. While the toxigenic culture and the cell cytotoxicity neutralization assay are still recognized as the gold standard for the diagnosis of CDI, new diagnostic approaches such as nucleic acid amplification methods have become available. In this review, we will discuss both current and emerging diagnostic and therapeutic modalities for CDI.

## Introduction

*Clostridium difficile* infection (CDI) has become a significant healthcare-associated infection with a considerable economic impact throughout the world and is particularly important in developed countries. In the United States alone, CDI is thought to cause approximately 453,000 infections and 29,000 deaths every year, with an annual economic burden ranging from $436 million to $3 billion dollars^1,2^. More worrisome is the increasing prevalence of fulminant *C. difficile* colitis in the past several decades^1,3^. This is due in part to newly recognized hypervirulent strains such as the *C. difficile* BI/NAP1/027 clone that expresses a binary toxin (CDT) in addition to the two large-molecule toxins TcdA and TcdB that are recognized as the primary virulence factors of CDI^2,3^.

Early detection of this pathogen and its toxins is critical as this allows earlier treatment that can significantly reduce the morbidity, mortality, medical cost, and family burden of CDI. The Food and Drug Administration (FDA) has approved a number of laboratory tests for the diagnosis of CDI. However, the epidemiology of CDI has drastically changed, with increasingly virulent strains emerging during the past decade^4^. An optimized and accurate diagnostic modality that can accurately differentiate CDI vs. colonization is urgently needed.

Antibiotic therapy remains the treatment of choice for CDI. While recommended antibiotics such as metronidazole, vancomycin, and fidaxomicin are still effective for this disease^1,5–7^, recent reports of *C. difficile* isolates with significantly reduced susceptibility and even resistance to these antibiotics suggest a potentially serious problem with the continued use of these agents to treat CDI^8,9^. Therefore, the development of new antibiotics and/or alternative treatment strategies as well as novel diagnostic approaches for CDI have become increasingly important. In this review, we will discuss both current and emerging diagnostic and therapeutic modalities for CDI.

## Current laboratory diagnosis

An effective diagnosis of CDI requires both clinical symptoms and a positive laboratory test^7,10^. A key clinical manifestation is diarrhea, which is defined as loose stools; this means taking the shape of the receptacle or corresponding to Bristol stool chart types 5–7, plus a stool frequency of at least three stools over 24 (or fewer) consecutive hours or more^7^. Diarrhea should be accompanied by abdominal pain as well as by systemic features such as fever, hypotension, and/or shock. Severe ileus in which diarrhea ceases, leukocytosis, and elevated serum creatinine levels are particularly important and should receive more attention^11^. Advanced age (≥65), marked leukocytosis (leukocyte count >15 × 10^9^/L), decreased blood albumin (<30 g/L), a rise in the serum creatinine level (≥133 µM or ≥1.5 times the premorbid level), and comorbidities (severe underlying disease and/or immunodeficiency) also should be regarded as poor prognostic markers for severe CDI^7^. In addition, antibiotic exposure should be assessed as most patients with CDI have had antibiotic exposure in the previous 3 months; any current antibiotic therapy should be discontinued if possible. When a patient exhibits loose stools that correspond to Bristol stool chart types 5–7 and has other risk factors for CDI in the absence of another obvious cause such as diarrheagenic medications or a different type of diarrheal illness, a fecal specimen should be collected for laboratory testing to assess the possibility of CDI. Except in case of paralytic ileus, formed stool samples should not be tested for CDI^10^.

Currently, there is no single stool test that can be relied upon as the reference standard for the diagnosis of CDI. Several methods are recommended for the diagnosis of CDI, including toxinogenic culture (TC), cell cytotoxicity neutralization assay (CCNA), enzyme immunoassays (EIA) for toxins A, B, and/or glutamate dehydrogenase (GDH), and nucleic acid amplification tests (NAATs)^7,10,12–16^. Among these methods, either the TC or CCNA has been considered to be the gold standard for the diagnosis of CDI over the past 30 years^17,18^. These tests use the following principles: TC detects the presence of *C. difficile* strains that actively produce toxin(s) (e.g., organism detection), whereas CCNA detects fecal protein toxins that have been produced in the stool (e.g., fecal toxin detection)^19–21^. It is noteworthy that both the TC and CCNA assays have limitations. These tests are labor intensive and have a slow turnaround time; thus, these tests are infrequently used for routine clinical diagnosis. TC has low specificity for clinical disease (CDI). The CCNA is analytically sensitive for toxin B, which historically has been considered a good marker for clinical disease. However, the performance of CCNA is dependent on pre-analytic factors and user experience and thus occasional clinical cases of CDI may be missed. Interpretation of these test results is subjective and requires some expertise; this may lead to poor reproducibility among different laboratories.

TC uses selective cycloserine–cefoxitin–fructose agar (CCFA) anaerobic culture and preliminary treatment with “heat shock” or “alcohol shock” in order to recover *C. difficile* from stool specimens; this method minimized the contaminating growth of other stool organisms. Suspect colonies are selected by presumptive tests, which include colony morphology, Gram stain, biochemical testing for indole (spot indole positive), and hydrolysis of L-proline-naphthylamide (“PRO Disk” positive). Presumptive identification of *C. difficile* is confirmed by RapID^TM^ ANA (Remel Products, Thermo Fisher Scientific) or matrix-assisted laser desorption ionization-time of flight mass spectrometry (MALDI-TOF MS). However, laboratory detection and identification of *C. difficile* alone does not diagnose CDI as 4% of healthy adults are colonized by *C. difficile*, and 20–25% of the *C. difficile* strains may be non-toxigenic^22^. Therefore, *C. difficile* isolates from positive TC tests should be evaluated for toxin production using CCNA and/or toxin EIA or evaluated by using NAAT to detect the presence of toxin A/B genes^10^.

Several commercial toxin-EIAs such as ProSpecT Toxin A/B (Remel Products, Thermo Fisher Scientific) and *C. difficile* Tox A/B II (TechLab, Inc.) as well as GDH-EIA tests such as C. Diff Chek-60 and C. Diff Quik Chek (TechLab, Inc.) have been introduced to the market. Overall, these products have a relatively low cost per test, but their sensitivity and specificity are not very good. The specificity of the toxin-EIAs varies widely, and sometimes their positive predictive values (PPVs) are inadequate for a diagnostic test^23^. While the GDH-EIA test methods are sensitive for screening *C. difficile* and also demonstrate a favorable negative predictive value (NPV)^23^, these methods are unable to differentiate toxigenic and non-toxigenic strains as both strains produce GDH^22^. Moreover, the GDH-EIA is not *C. difficile*-specific due to cross-reaction with similar enzymes yielded by other clostridial species^24^. Therefore, EIAs combining GDH-EIA and toxin-EIA in one test have been developed. In 2009, TechLab, Inc. developed the C. Diff Quik Chek Complete to detect GDH and toxin A/B simultaneously. This combination test provided a rapid, cost-effective, and easy method for diagnosing CDI. With a 98% specificity and the results being available in 30 min, a negative result using the GDH-EIA can rule out CDI without the need for additional tests.

The use of NAATs for the detection of *C. difficile* from diarrheal stool specimens was documented in the early 1990s^23^. NAATs possess a series of advantages such as excellent sensitivity and specificity, low complexity, simplified reporting, reduced need for repeat testing, and improved turnaround time. It has been noted that the sensitivity of GDH screening tests for *C. difficile* is lower than that using NAATs, and NAATs for *C. difficile* toxin genes are superior to toxin-EIA testing as a standard diagnostic test for CDI^24^. Accordingly, NAATs are regarded as the most cost-effective method for the diagnosis of CDI^25^. Most NAATs target the encoding genes of TcdB, TcdA, and/or the binary toxin^26–28^. In particular, some NAATs such as multiplex NAATs can simultaneously detect *C. difficile* strains and toxin encoding genes from stool samples^29^. There are several commercially available NAATs, including a real-time PCR (RT-PCR) assay and loop-mediated isothermal amplification (LAMP) assay, both of which have an overall high analytical sensitivity (80–100%) and specificity (87–99%)^30^. In addition, several multiplex NAATs panels/platforms have been developed to detect stool pathogens including *C. difficile* from stool specimens. The first one is the Luminex xTAG GPP (Luminex Molecular Diagnostics Inc.), which targets 11 different stool pathogens (7 bacterial, 2 viruses, and 2 parasites); the second one is FilmArray™ Gastrointestinal Panel (BioFire Diagnostics), which is based on nested multiplex PCR and detects 23 stool pathogens (14 bacteria, 5 viruses, and 4 parasites)^30,31^. The sensitivities and specificities of these two assays for the detection of *C. difficile* were 91% and 100%, and 95% and 99%, respectively^32,33^. Moreover, multiplex NAATs can rule out diarrhea caused by other gastrointestinal pathogens even though *C. difficile* has been the most frequently detected pathogen in diarrheal disease. It is currently difficult to assess this novel syndromic approach to gastroenteritis due to its costs, insufficient data, and limited value in hospitalized patients. There are also some limitations with NAATs. These molecular methods do not differentiate between active toxin production in vivo and *C. difficile* colonization without toxin production^22^ because NAATs only detect toxin encoding genes rather than directly detecting the toxin in stool. Thus, these molecular methods are unable to distinguish the CDI from colonization in patients with other reasons for a diarrheal illness, which may result in the overdiagnosis of CDI^34^. Therefore, additional tests such as an EIA test for *C. difficile* toxin A and/or toxin B may be needed to confirm the presence of toxin production in vivo and thus establish the likelihood of CDI and the need for treatment^11,22^. The final confirmation of CDI may require a combination of these tests along with the presence of clinical symptoms and signs.

**Fig. 1:**
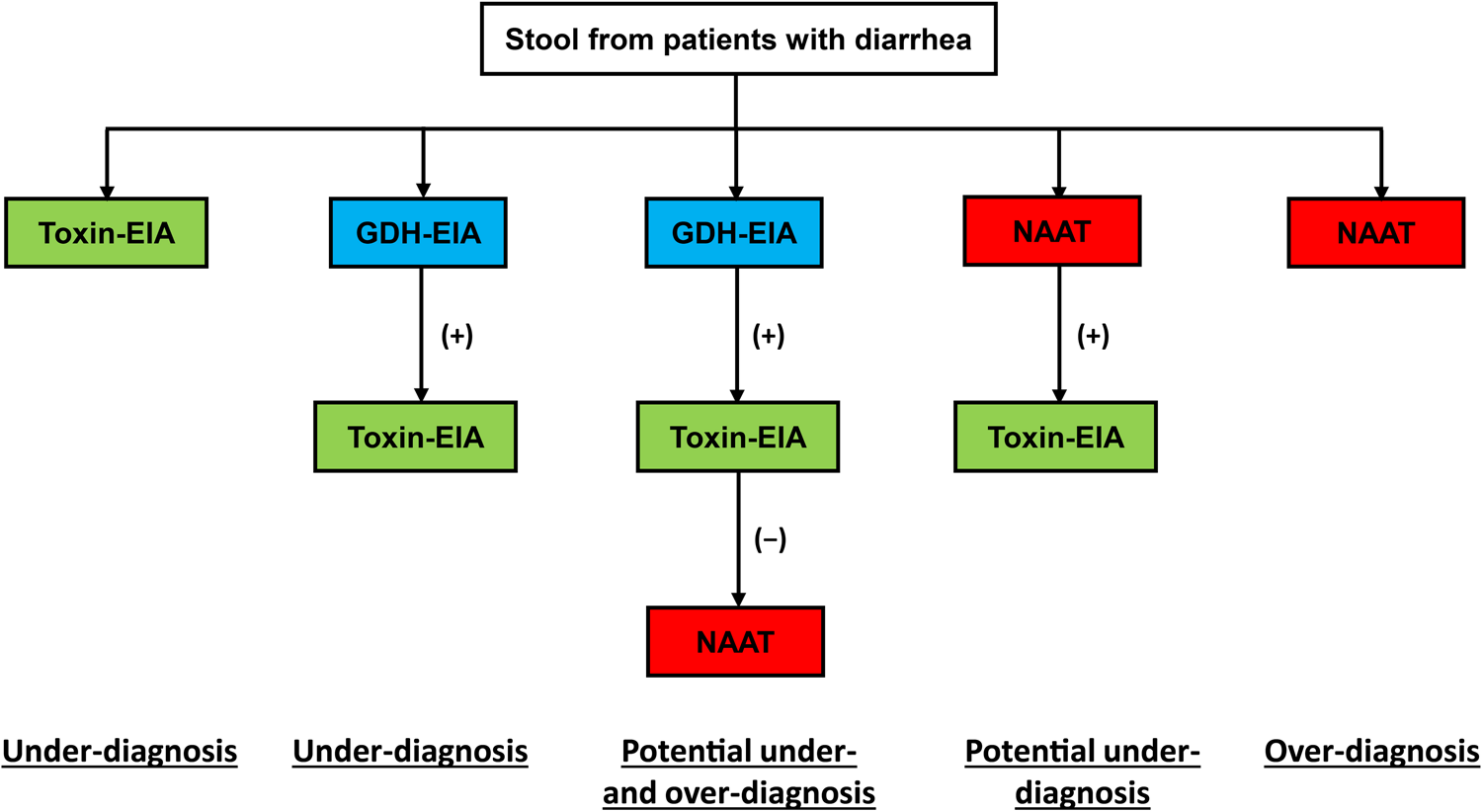
Test algorithms for the diagnosis of *Clostridium difficile* infection. EIA enzyme immunoassay, GDH glutamate dehydrogenase, NAAT nucleic acid amplification test, (+) positive, (−) negative

Although NAAT methods are considered to be superior to other methods of diagnosing CDI, this testing strategy is unable to accurately distinguish between *C. difficile* colonization and disease^22^, which sometimes results in overdiagnosis of CDI^35^. Such overdiagnosis could result in overtreatment of CDI, delayed recognition of other causes of diarrheal illness/outbreaks, unnecessary exposure of antibiotics used to treat CDI, and overestimation of hospital CDI rates^36^. Several test algorithms have been developed to improve the rapid and accurate diagnosis of CDI (Fig. 1). The European Society of Clinical Microbiology and Infectious Diseases (ESCMID) recommends the use of a two-step algorithm starting with either NAATs or GDH-EIA tests. Samples with a negative result from either NAATs or GDH-EIA tests can be reported as CDI negative, but those having a positive result should be confirmed by a toxin-EIA. Samples confirmed by this second toxin-EIA test can be reported as CDI positive^10^. This two-step algorithm may help reduce NAAT-related overdiagnosis of CDI.

**Fig. 2:**
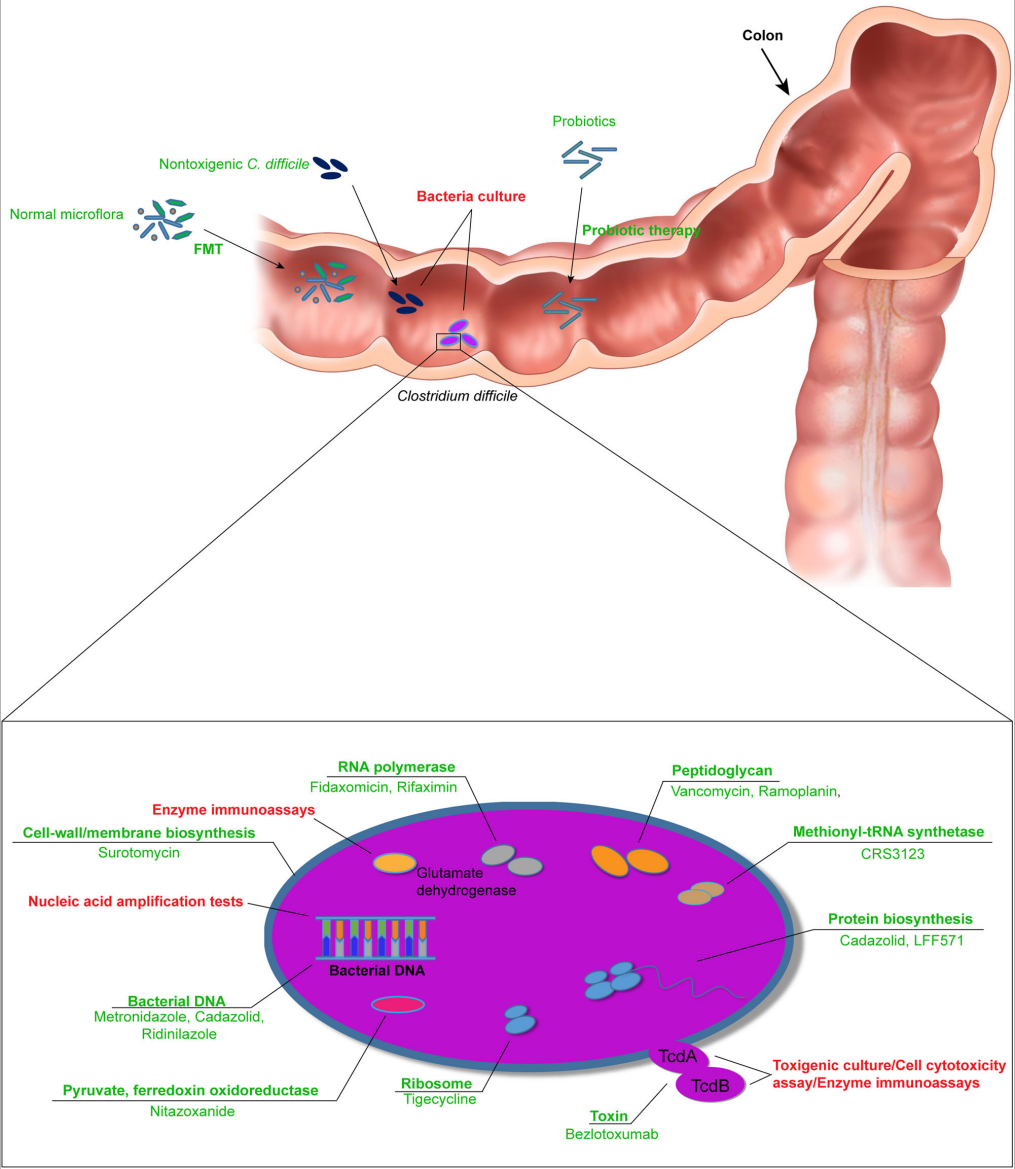
Bacterial targets for CDI therapeutics. Texts in red and green are the indicated targets for diagnostic and therapeutic approaches, respectively

Strategies such as intervention on the frequent use of PCR testing and/or using a modified NAAT cutoff to predict toxins also are options for reducing inappropriate testing^36–39^. Because *C. difficile* PCR is a very sensitive method and frequently identifies colonized patients, the frequent use of PCR testing in low-risk patients may lead to the misdiagnosis of CDI. Therefore, highly sensitive PCR testing should not be ordered in patients with a low probability of infection (i.e., a patient without risk factors who has vomiting as a presenting complaint; a patient has a soft or formed stool; a patient with diarrhea who is using stool softeners or laxatives)^36^. In addition, quantitating *C. difficile* concentration in stool samples using RT-PCR can help to distinguish patients with CDI from the patients colonized with *C. difficile* who have diarrhea due to other causes, yet are still recognized as carriers of *C. difficile* requiring prevention of transmission^37^.

In certain clinical settings, adjunct testing methods such as radiologic diagnostic imaging may be useful for diagnosing CDI. Diagnostic computed tomography (CT) imaging can assist with an early diagnosis and also may determine the severity of the disease in patients with CDI^2^. For example, patients with ileus can be identified by clinical symptoms of severely disturbed bowel function such as vomiting and absence of bowel movements along with radiological signs of bowel distension. Patients with toxic megacolon can be identified by clinical signs of a severe systemic inflammatory response along with radiological signs of colon distension (>6 cm for the transverse width of the colon)^7^. However, it should be noted that the radiological diagnostic methods generally provide little value for the diagnosis of CDI^40^.

## Strain typing

Strain typing also may be an important component of the laboratory diagnosis of CDI. In particular, ESCMID recommends strain typing of isolates recovered from the CDI case in an outbreak situation^10^. Accurate methods for strain characterization also are required for distinguishing circulating strains, new strains, and emerging pathogenic strain variants. There are several molecular typing methods available for the laboratory typing of *C. difficile*. Pulsed-field gel electrophoresis (PFGE) was one of the first typing methods used for the molecular analysis of *C. difficile* strains in North America. However, this method cannot accurately separate very large molecules of DNA and thus can result in subjectivity for the interpretation of the results, especially when there are subtle differences between the target strains and the reference strains^23^. In Europe, ribotyping (RT) has become the most frequently used method for *C. difficile* typing. This method demonstrates a good discriminatory ability, despite some difficulty in differentiating the closely related strains such as RT 027, 106, and 017^41,42^. Multilocus sequence typing (MLST) was used for the first time for *C. difficile* typing in 2004^43,44^. The method is particularly useful for typing *C. difficile* strains from stool specimen owing to the genetically heterogeneous characteristics of such strains. Mutilocus variable-number tandem-repeat analysis (MLVA) is a discriminatory molecular subtyping method based on capillary electrophoresis that can evaluate seven or eight loci on the *C. difficile* genome that also has proven useful for *C. difficile* strain typing. MLVA has also been used in outbreaks to identify phylogenetic strains as well as for performing surveillance for transmission^23,41,45^.

The epidemiology of CDI has drastically changed over the past several decades due to the emergence of highly virulent stains, which demonstrates the capability of *C. difficile* strains in general to become more virulent^4^. Therefore, it is likely that additional new epidemic strains of *C. difficile* will emerge and will require typing. The assessment of hospital-wide transmission with the use of MLST or RT has been hampered by the large numbers of patients who share a genotype and hospital-based contact. Whole-genome sequencing (WGS) shows that substantial genetic diversity exists among these strains, even within isolates of the same genotype^46^. In 2012, WGS using an Illumina platform was used for investigating the dissemination of *C. difficile* in the United Kingdom^47^. A previous study that defined the global demographic structure of *C. difficile* 027/BI/NAP1 using WGS and phylogenetic analysis has noted two distinct lineages (FQR1, FQR2) for the epidemic strain in North America. This approach has been able to track the spread of this strain throughout the world^48^. WGS is a powerful genotyping tool and has been used to quantify the role of symptomatic patients in *C. difficile* transmission^49^, to track the transmission of individual clones in infected hospital patients^50^, and to identify varying rates of *C. difficile* transmission between institutions^51^. However, high cost and a slower turnaround time as well as the challenge of a large data file that requires a suitable bioinformatics system for analysis are several concerns regarding WGS.

## Current antibiotics for the therapy of CDI

Antimicrobial therapy remains the first choice for CDI, and specific antimicrobial therapy guideline recommendations should be based on the severity of the disease. Therapy of CDI should include both cessation of the inciting antimicrobial agent as soon as possible as well as implementation of infection control measures. If continued antimicrobial therapy is required for the treatment of the primary infection, antimicrobial therapy with agents that are less frequently implicated in antibiotic-associated CDI should be used; these include parenteral aminoglycosides, sulfonamides, macrolides, vancomycin, or tetracycline/tigecycline (Fig. 2). Supportive care such as correction of fluid losses and electrolyte balance also should be addressed. Finally, the severity of CDI should be assessed as the first step of therapy. There is no consensus definition for severe CDI, nor which clinical indicators should be considered when defining severity. Current guidelines suggest that a serum albumin <3 g/dL plus either a white blood cell count ≥15,000 cells/mm^3^ or abdominal tenderness without additional criteria for complicated disease are important clinical indicators that suggest severe CDI^24^. In addition, age >60 years, temperature ≥38.5 °C, endoscopic evidence of pseudomembranous colitis, and admission to the intensive care unit are also useful clinical indicators that suggest severe CDI. A fulminant or complicated course is suggested by shock, megacolon, perforation, need for colectomy, as well as older age, high leukocyte count, and acute renal failure^24^. Moreover, there are relatively few classes of antimicrobial agents that are currently recommended for the therapy of CDI; these include the imidazoles (e.g., metronidazole), glycopeptides (e.g., vancomycin), and macrolides (e.g., fidaxomicin)^5–7^. Both metronidazole and vancomycin have been used for more than 30 years and remain the drugs of first choice^1,2,11^.

## Severe, complicated, or fulminant cases of CDI

A combination of metronidazole (500 mg intravenously three times a day for 10–14 days) and vancomycin (125 mg orally four times a day for 10–14 days) is recommended for severe, complicated, or fulminant cases of CDI^1,11,24^. For patients with severe CDI who do not respond within 24–48 h or who develop complications such as renal failure or ileus, the oral dose of vancomycin should be increase to 500 mg four times a day. The use of additional vancomycin therapy via rectal retention enema, 500 mg in 100 mL normal saline every 6 h, is recommended if complete ileus is present. Fidaxomicin may be considered for patients who cannot tolerate vancomycin; the dose is 200 mg orally twice per day. Colectomy or ileostomy is still a useful adjunctive therapy for fulminant cases^1,11,24^. Close monitoring and supportive care is required for severe, complicated, or fulminant cases of CDI; surgery should be considered if the patient’s clinical status is not improving and/or the serum lactate is above 2.2 mmol/L. Consider toxic megacolon if the patient develops abdominal distention with lessening of diarrhea, which strongly suggests paralytic ileus due to loss of colonic muscular tone.

## Mild-to-moderate cases of CDI

Although oral metronidazole 500 mg three times per day for 10–14 days is still recommended for mild-to-moderate cases of CDI (defined by white blood cell count higher than 15,000 cells/mm^3^ and blood urea nitrogen or creatinine levels above baseline)^1,11,24^, metronidazole is associated with more frequent side effects, and there has been a significant increase in treatment failures (especially in patients infected with the emergent 027/BI/NAP1 strain).

Recent data have suggested an overall superiority of vancomycin to metronidazole for the treatment of patients with mild and moderate CDI^1^. However, vancomycin treatment is significantly more expensive than metronidazole. Oral vancomycin 125 mg four times per day for 10–14 days is recommended as an alternative for moderate cases^1,11^. For less severe cases, oral vancomycin 125 mg four times per day for 10–14 days is preferred, but oral fidaxomicin 200 mg twice a day for 10 days is also recommended if the risk of recurrence is high. Fidaxomicin is considered to have a similar therapeutic efficacy as oral vancomycin with a significantly lower recurrence rate^1,11^.

## Recurrent cases of CDI

For recurrent cases of CDI, oral vancomycin 125 mg four times per day for 14 days or oral fidaxomicin 200 mg twice a day for 10 days is recommended for first recurrence. Vancomycin in a tapered and pulsed regimen, fecal microbial transplantation, or fidaxomicin 200 mg twice a day for 10 days is recommended for second or further recurrences^1,24^.

In addition to metronidazole, vancomycin, and fidaxomicin, other antibiotics such as rifaximin, nitazoxanide, ramoplanin, teicoplanin, and tigecycline have been used for cases where unacceptable adverse effects have been associated with standard therapy^1^. These antibiotics also have been used when there is need for salvage therapy due to fulminant disease, and surgery is not possible, as well as for the therapy of intractable recurrent infection^1^. However, these antibiotics are not recommended as drugs of first choice due to limited data, high cost, an unfavorable adverse-event profile, and/or resistance to *C. difficile*^1^.

## Emerging antibiotics

There are a number of antibiotics that are currently being evaluated for the therapy of CDI. These agents are in different phases of clinical trials (Table 1). Among them, two unique types of antibiotics, cadazolid (developed by Actelion) and surotomycin (developed by Merck) have completed phase III clinical studies, while another two types of antibiotics, LFF571 (Novartis) and Ridinilazole (formerly SMT19969, Summit Pharmaceuticals), have completed phase II studies, and one antibiotic, CRS3123 (National Institute of Allergy and Infectious Diseases), is being evaluated in phase I studies^52^.

**Table 1:** Emerging antibiotics evaluated in clinical trials that display inhibition against Clostridium difficile*

Antibiotics	Targets	Mechanism	Investigation phase
Inhibitors of protein synthesis			
LFF571	Elongation factor Tu	Inhibits the delivery of aminoacyl-tRNA to the ribosome	Phase II completed
CRS3123	Methionyl-tRNA synthetase	Inhibits *C. difficile* methionyl-tRNA synthetase	Phase I ongoing (NCT02106338)
Agents with direct effect on cell wall/cell membrane			
Surotomycin	Cell membrane		Phase III completed (NCT01597505)
Hybrid antibiotics and other agents with multiple mechanisms of action			
Cadazolid	50S ribosome subunit and topoisomerase	Inhibits protein synthesis + interferes with DNA synthesis	Phase III ongoing (NCT01987895)
Agents of unknown/unclear mechanism of action			
Ridinilazole (SMT19969)			Phase II completed (NCT02092935)

^*^ Adopted from Fehér et al.^52^

Cadazolid is a novel hybrid of oxazolidinone that displays a potent antimicrobial activity against *C. difficile* and has a lower propensity for inducing resistance^53^. In previous in vitro and in vivo antimicrobial evaluation studies, cadazolid was found to be more bactericidal than vancomycin. Moreover, cadazolid strongly inhibited de novo toxin A and B formation in stationary-phase cultures of toxigenic *C. difficile* and also inhibited *C. difficile* spore formation substantially at growth-inhibitory concentrations. In particular, cadazolid was active with a potency similar to that of vancomycin in conferring full protection of hamster and mouse models from diarrhea and death caused by *C. difficile*^54^. Another in vitro and in vivo study demonstrated that cadazolid had a stronger inhibitory effect on *C. difficile* in vivo than did moxifloxacin, linezolid, metronidazole, and vancomycin^55^. Finally, cadazolid more effectively treated CDI in a simulated gut model and had limited impact on the enumerated gut microflora and no signs of recurrence or emergence of resistance within the experimental timeframe^55^. Data from a multicentre, double-blind, phase 2 study of oral cadazolid in patients with CDI demonstrated a lower recurrence rate in patients that received 250, 500, or 1000 mg cadazolid twice daily than those receiving 125 mg vancomycin four times daily (18.2–25.0% vs. 50%)^56^. The patients treated with cadazolid had higher sustained clinical response rates than those treated with vancomycin (46.7–60.0% vs. 33.3%)^56^. A following study noted that cadazolid was more effective against the *C. difficile* strains isolated from the patients in this phase 2 study because the minimum inhibitory concentrations (MICs) for cadazolid were lower than those of vancomycin, linezolid, and moxifloxacin^57^. Cadazolid is now being evaluated in phase 3 clinical trials^52^; the data from these studies are not yet available.

Surotomycin is an orally dosed, non-absorbed cyclic lipopeptide analog of daptomycin^58^. Surotomycin works through a mechanism that disrupts *C. difficile* cellular membrane activity in both logarithmic and stationary phases. Surotomycin is minimally disturbing to the normal gastrointestinal microbiota because of its lack of activity against Gram-negative anaerobes and facultative anaerobes. This antibiotic has been successful in reducing *C. difficile* vegetative cell counts and toxin levels in chemostat gut models^59^. A phase 2 clinical trial has shown that oral surotomycin at 125 mg twice daily or surotomycin 250 mg twice daily for 10 days resulted in a similar clinical cure rate (92.4% and 86.6%) to that seen with vancomycin 125 mg four times daily for 10 days (89.4%), but the recurrence rates of the surotomycin treatment groups (27.9% for surotomycin 125 mg twice daily and 17.2% for surotomycin 250 mg twice daily) are lower than the vancomycin treatment group (35.6%)^60^. More importantly, the sustained clinical response rates seen in this trial for the surotomycin groups (66.7% for surotomycin 125 mg twice daily, 70.1% for surotomycin 250 mg twice daily) at the end of study were higher than the vancomycin group (56.1%)^60^. In addition, a phase 3 clinical trial of surotomycin in subjects with CDI has been completed^60^. In this study, subjects with CDI confirmed by a positive toxin result were randomized to receive surotomycin (250 mg twice daily) or vancomycin (125 mg 4 times daily) orally for 10 days. However, the initial data from this trial suggest that surotomycin failed to meet the criteria for non-inferiority compared with vancomycin for the primary and key secondary endpoints. In this trial, both the clinical cure rate and sustained clinical response rate of the subjects treated with surotomycin were lower than those who received vancomycin treatment (clinical cure rate: surotomycin 79.0% vs. vancomycin 83.6%; sustained clinical response rate: surotomycin 60.6% vs. vancomycin 61.4%)^60^.

LFF571 is a semisynthetic derivative of GE2270A and works through inhibiting bacterial protein synthesis by interacting with elongation factor Tu (EF-Tu) and interrupting complex formation between EF-Tu and aminoacyl-tRNA^61,62^. It has been noted that LFF571 has an excellent activity against *C. difficile* as well as a good activity against other Gram-positive anaerobes, but little activity against the anaerobic Gram-negative organisms^63^. This drug was found to be more efficacious in the hamster model of CDI than vancomycin; it had a lower effective dose and fewer recurrences^64^. The safety and tolerability of LFF571 in humans has been assessed in a randomized, double-blind, placebo-controlled phase I trial, which demonstrated that the drug was generally safe and well tolerated in single and multiple oral doses in healthy volunteers^65^. Now a phase II trial comparing the safety and efficacy of LFF571 and vancomycin for CDI has been completed; its results suggest that LFF571 treatment is non-inferior to vancomycin treatment. The incidence of adverse events related to LFF571 in this study was higher than that related to vancomycin (76.1% vs. 69.2%)^66^. However, the true incidence of adverse events related to LFF571 needs to be further investigated as the study was limited by a small enrollment. It should be noted that LFF571 has a role in reducing *C. difficile* toxins. A recent study found that an in vitro treatment with LFF571 led to a reduction in toxin A and B production from various *C. difficile* strains^62^.

Ridinilazole, formerly known as SMT19969, is a novel, narrow-spectrum, non-absorbable small molecule antimicrobial with activity against *C. difficile*^67^. However, the detailed mechanism of action of this drug remains unclear. This agent does not appear to act through the usual mechanisms associated with antibiotics, such as inhibition of cell wall, protein, lipid, RNA or DNA synthesis^68^. A recent study found that ridinilazole had a robust killing effect on *C. difficile*, which significantly reduced toxin production and attenuated the inflammatory response^67^. Ridinilazole also elicited significant cell division effects^67^. Those findings suggest a potential mechanism of action. The antimicrobial activity of ridinilazole has been well studied. A comparative in vitro study of ridinilazole against *C. difficile* found that ridinilazole was more active against *C. difficile* isolates including the 027/BI/NAP1 strains than fidaxomicin, vancomycin, and metronidazole^69^. Ridinilazole also showed limited activity against other members of the Gram-positive anaerobic intestinal flora^69^. A phase 2, randomized, double-blind, active-controlled, non-inferiority study of ridinilazole has been completed; results from this study demonstrated that ridinilazole was well tolerated and had an adverse event profile similar to that of vancomycin^70^. Moreover, data from this trial established the non-inferiority of ridinilazole to vancomycin, as patients in the ridinilazole group had a higher sustained clinical response than those in the vancomycin group. Finally, ridinilazole showed statistical superiority at the 10% level in comparison to vancomycin.

CRS3123 (Crestone Inc., Johnston, CO) is a small molecule narrow-spectrum agent with Gram-positive coverage and limited oral bioavailability, whose use results in a high concentration of the drug in the gastrointestinal tract and low systemic exposure^71^. This agent is a novel aminoacyl-tRNA synthetase inhibitor. It has been proposed that this unique antibiotic has a role in inhibiting both the growth, spore formation, and toxin production of *C. difficile*^71,72^. CRS3123 has been shown to be active against all *C. difficile* clinical strains tested, including the epidemic 027/BI/NAP1 strains. Treatment of CDI in the hamster model using oral CRS3123 has long-lasting efficacy with no recurrence. In addition, this agent does not display cross-resistance to existing antibiotics and remains active to the tested *C. difficile* strains^72^. CRS3123 is currently investigated in early-stage clinical trials.

## Alternative treatment strategies

Since the expression of clostridial toxins (TcdA and TcdB) is mandatory for the development of CDI, the development of agents such as monoclonal antibodies aimed at preventing the cytotoxic effect of these toxins is a sensible strategy for controlling the disease. In 2016, the US FDA approved Merck’s ZINPLAVA™ (bezlotoxumab) to reduce the recurrence of CDI in adult patients receiving antimicrobial therapy for CDI who are at high risk of CDI recurrence (http://www.mercknewsroom.com/news). Bezlotoxumab (MK-6072) is a human monoclonal antibody which reduces recurrent CDI by blocking the binding of *C. difficile* toxin B to host cells and therefore limiting epithelial damage and facilitating microbiome recovery^73,74^. The data from two separate phase 3 trials comparing bezlotoxumab with placebo among participants who were receiving standard-of-care therapy with oral vancomycin, metronidazole, or fidaxomicin showed that bezlotoxumab achieved a significant benefit over placebo^74,75^. Both the bezlotoxumab regimen and the actoxumab–bezlotoxumab regimen had good safety profiles without substantial adverse reactions; diarrhea and nausea were the most common adverse events^74,75^. The rate of recurrent infection in one of the phase 3 clinical trials was 17% vs. 28%, favoring bezlotoxumab (*P* < 0.001); and the rate in the another phase 3 clinical trial was 16% vs. 26%, also favoring bezlotoxumab (*P* < 0.001)^74,75^. Besides bezlotoxumab, Merck also developed another human monoclonal antibody, actoxumab (MK-3415), which is designed to neutralize *C. difficile* toxin A^75^. Although the data from human clinical trials that evaluate actoxumab alone are still limited, a phase 3 study found that a significantly lower rate of recurrent CDI (15.4%) was observed in the group of combination use of actoxumab and bezlotoxumab vs. the group using placebo (26.6%, both 1-sided, *P* < 0.0001), and global cure was slightly higher for the combination of actoxumab and bezlotoxumab (58.1%, 1-sided *P* = 0.0426) vs. placebo (53.7%)^74,75^. In addition, a combination of actoxumab and bezlotoxumab was found to have an ability to facilitate normalization of the gut microbiota in CDI mice^76^.

Effective vaccines against CDI are not yet available, but several promising vaccine candidates are being studied. According to information on the National Institutes of Health website ClinicalTrials.gov, there are three *C. difficile* vaccines currently in different stages of clinical trials: Cdiffense in phase III (NCT01887912), which is a vaccine containing toxoids of TcdA and TcdB from Sanofi Pasteur; IC84 in phase II (NCT02316470), which is a vaccine consisting of recombinant protein of the two truncated toxins TcdA and TcdB from Valneva, and a bivalent toxin vaccine from Pfizer in phase II (NCT02561195, NCT02117570). All three vaccine candidates target TcdA and TcdB. In addition, there are other vaccines being developed; these include recombinant vaccines based on the polysaccharide glycans, glycoconjugate vaccines, and DNA-based vaccines^52,71,77^. Many of these vaccines have displayed good efficacy for CDI under laboratory conditions or in clinic trials.

Fecal microbiota transplantation (FMT) is designed to restore the normal gut microflora for patients with CDI. This method has been used for more than 1000 years, but has only been recognized as a valid and effective method for the therapy of multiple recurrent CDI since 2010^78^. The use of FMT to treat recurrent/relapsing cases of CDI has proven to be safe and effective; its advantage over antimicrobial therapy is now being widely lauded^72^. Previous reviews of FMT have documented that oral or rectal transplantation of feces from a healthy, pretested donor combined with the simultaneous cessation of all antimicrobial use in the recipient is successful in treating >90% of patients with recurrent CDI^1,79^. Moreover, these reviews have found that the administration of vancomycin followed by an infusion of donor feces delivered by a nasoduodenal tube was safe and superior to vancomycin alone for recurrent CDI^1^. Particularly, FMT is now recommended as the therapeutic option of choice if there is a third recurrence after a pulsed vancomycin regimen^24^. However, the potential role of FMT in primary CDI is still not well understood.

Donor screening should be considered as the most important factor for FMT. When a proper donor is decided, his/her feces are handled via a series of processes including dilution of the specimen with normal saline (not the only choice of diluent but that which is generally used), homogenization, and filtration for direct transplantation or frozen for future use^80^. Routes of FMT administration include the upper gastrointestinal tract (with endoscopy, nasointestinal tubes, or pill ingestion), the proximal part of the colon by colonoscopy, or the distal part of the colon by enema, rectal tube, or sigmoidoscopy. A method that combines several of these methods of administration may be preferable in complex cases^78^. A recent study noted that fecal samples delivered via the lower gastrointestinal tract is a safe and effective treatment for refractory and recurrent CDI, and yields quicker results than delivering through upper gastrointestinal tract^81^. It should be pointed out that there is no FDA indication for FMT. Therefore, FMT for CDI can be performed only with an Investigational New Drug (IND) approval if the providers follow general ethical guidelines^1,78^. Moreover, it has been suggested that practitioners not having an experience in FMT first should consult FDA guidelines before performing FMT^78^. It also should be pointed out that the safety profile of FMT has not been well studied. Common postprocedural symptoms after FMT include abdominal pain, bloating, flatulence with borborygmus, diarrhea, constipation, vomiting, transient fever, and belching; these symptoms are usually transient and resolve within a few hours^78^.

Although FMT has an 80–95% success rate with long-term durability^81^, a number of disadvantages still exist. In particular, the manipulation of feces and the classical enteral administration methods are not only laborious, but tend to make the procedure rather unattractive for physicians and patients alike^52^. With regard to these disadvantages, a number of efforts have been made to enhance the feasibility and social acceptance of microbiota transplantation. Oral administration of capsulized intestinal flora is a good example of such efforts. Another example is the commercial application of intestinal microbiota restoration using live encapsulated microorganisms that are taken orally by the patient. On July 29, 2013, the US FDA has approved Rebiotix Inc.’s (Roseville, Minnesota) IND application to begin the phase 2 clinical study of RBX2660 for the treatment of recurrent CDI. RBX2660 is a commercially prepared, standardized, next-generation FMT live biotherapeutic product that initially was administered by enema for recurrent CDI. The data from the first of two phase II studies showed an overall 87.1% success rate of using RBX2660 to treat CDI^82^. SER-109 (Seres) is another live biotherapeutic that comprises an encapsulated mixture of purified *Firmicutes* spores, derived from human feces^83^. Two phase II studies of SER-109 have already been completed, and their results indicated that SER-109 was not effective overall at reducing CDI recurrence, but was efficacious in patients aged at least 65 years old^83^.

Probiotic therapy is another strategy for restoring the colonic microbiota that has been investigated for many years as a therapy to treat or prevent CDI. A study performed at Xinhua/Yuyao Hospital in Shanghai, China, has demonstrated that the use of a probiotic combination containing *Lactobacillus acidophilus* CL1285 and *Lactobacillus casei* LBC80R (Bio-K+) was well tolerated and effective in reducing the risk of antibiotic-associated diarrhea (AAD), including *C. difficile*-associated diarrhea (CDAD), in hospitalized patients on antibiotics^84^. Another observational study performed at a community hospital in Quebec, Canada, provides additional support. In this study, all adult inpatients on antibiotics in the community hospital Pierre-Le Gardeur (PLGH) between 30 April 2004 and 31 March 2014 were given a probiotic mixture containing *L. acidophilus* CL1285, *L. casei* LBC80R, and *Lactobacillus rhamnosus* CLR2 (Bio-K+) within 12 h of receiving an antibiotic prescription. During the 10 years of observation, 44,835 inpatients received the probiotic, and the CDI rate declined from 18.0 cases per 10,000 patient-days to an average of 2.3 cases per 10,000 patient-days^85^. In the same study, 10-year data revealed that the CDI rates at PLGH were consistently and continuously lower compared with those at similar hospitals in Quebec^85^. Moreover, a number of systematic reviews of probiotics have suggested that probiotics are safe and may be effective in treating and preventing CDAD^86,87^. Finally, a recent study has found that a combination of probiotic and prebiotic products were more effective in preventing the germination of *C. difficile* spores and thus preventing CDI^88^. However, it should be noted that the evidence for the use of adjunct probiotics to decrease recurrences in patients with recurrent CDI is still limited. For example, another series of studies found no benefit with probiotic administration in the prevention of CDI^89,90,91,92,93^; currently probiotic therapy is not recommended for the therapy of CDI due to the limited data and potential risk of bloodstream infection^2^.

In addition to FMT and probiotic therapy, minimizing the disruption of the normal gut microflora should be also considered. Some bacteriocins such as thuricin CD, nisin and lacticin 3147, NVB302, and GE2270 derivative have demonstrated selective and potent inhibition of *C. difficile* and other Gram-positive bacteria, but have little or no impact on other commensal gut microbes^77^. Other antimicrobial agents such as MGB-BP-3, OPS-2071, Av-CD291.2, lauric acid (derived from virgin coconut oil), berberine, and bovine lactoferrin also have been described in a recent review that systematically summarizes the experimental and off-label therapies for CDI^52^. While further studies are still required, many of those emerging agents might have a potential application for the prevention or treatment of CDI.

The administration of non-toxigenic *C. difficile* strains is another approach to prevent the colonization of the intestinal microbiota by toxigenic *C. difficile*. Much like FMT, this strategy uses non-toxin-producing *C. difficile* to colonize the gastrointestinal tract and thus prevent colonization by toxin-producing strains; this strategy has been shown to alleviate CDI in both patients and animal models^94,95,96,97^. Moreover, studies using animal models have demonstrated that the administration of non-toxigenic *C. difficile* strains provided good protection to hamsters and/or mice against a challenge with the hypervirulent BI/NAP1/027 *C. difficile*^95,96^. In a more recent phase II trial, colonization with the non-toxigenic *C. difficile* was found to correlate with reduced recurrence of CDI; recurrence of CDI in patients purposefully colonized with non-toxigenic *C. difficile* was 2% compared with 31% in patients who did not receive non-toxigenic *C. difficile* in order to colonize their intestinal microbiota^97^. Another commercial product, VP20621 developed by Shire, is composed of a non-toxigenic strain M3 and has finished a phase II clinical trial in 2013 (NCT01259726)^94^. This trial has demonstrated that the oral administration of non-toxigenic *C. difficile* strain M3 spores was well tolerated and safe; the non-toxigenic strain colonized the gastrointestinal tract and significantly reduced CDI recurrence^94^. It should be noted that although the results of this study are positive, the use of non-toxigenic *C. difficile* for the therapy of CDI is still thought to be questionable as the non-toxigenic *C. difficile* strains might acquire the known pathogenicity locus that encodes the *C. difficile* toxins from toxigenic strains via horizontal gene transfer and thus become toxin producers^98^.

Surgical consultation is recommended for all patients with complicated CDI. Such consultation should be considered for patients having any of the following symptoms/signs: hypotension with or without required use of vasopressors, fever ≥38.5 °C, ileus or significant abdominal distention, mental status changes, WBC ≥35,000 cells/mm^3^ or <2000 cells/mm^3^, serum lactate levels >2.2 mmol/L, and/or end organ failure (mechanical ventilation, renal failure, etc.)^32^. Complicated CDI with failure to improve on medical therapy after 5 days also requires surgical consultation^32^. For cases of CDI, the more negative prognostic signs a patient has, the earlier operative management should be considered as surgery is more likely to improve the survival when done in a timely manner.

## Emerging strategies for the therapy of CDI

Due to the continued development of science and technology, a number of non-antibiotic therapeutics are emerging that may be useful for the therapy of CDI. While most of these therapeutics are still in the laboratory phase, their emergence might provide future therapies for the treatment of CDI. The potential use of bacteriophage lysin proteins is one of such emerging strategies. A bacteriophage lysin protein and its catalytic domain (PlyCD_1-174_) cloned from the prophage sequence harbored in *C. difficile* CD630 genome has demonstrated an excellent capability for lysing *C. difficile*^99^. Indeed, the catalytic domain (PlyCD_1-174_) was found to have a broader lytic spectrum against this pathogen^99^. In addition, subinhibitory doses of vancomycin combined with the PlyCD catalytic domain in vitro were significantly more bactericidal against *C. difficile* than was vancomycin alone^99^.

The human alpha-defensins HNP-1, HNP-3, and HD-5 also have been reported to have a potential role in preventing the cytotoxic effects of *C. difficile* toxin B in intestinal epithelial cells and in a large array of other cells^100^. This suggests a defense mechanism for human defensins against some types of clostridial glucosylating cytotoxins^100^. A similar study found that both HNP-1 and HD-5 also displayed good killing effects against *C. difficile*, with HD-5 having a significant bactericidal activity against hypervirulent *C. difficile* strains^101^. It is suggested that HD-5 used in combination with FMT therapies would be useful for treating recurrent forms of CID due to the antitoxin, bactericidal, and immunostimulatory effects of such a treatment^101^.

Another more recent study has described a synthesized, bioactive, low molecular weight organoselenium compound, ebselen, that directly targets the glucosyltransferase domain (GDT) of *C. difficile* toxins. This agent was found to have a good activity against both TcdA and TcdB^102^, and in treatment tests in a mouse model that closely resembles human infection, treatment with ebselen reduced the disease pathology in murine tissues by inhibiting the release of the toxic GDT^102^.

Tolevamer is a non-absorbable high molecular weight anionic polymer that could potentially absorb the toxins (TcsA and TcdB) involved in CDI. Clinical trials evaluating tolevamer for the therapy of CDI have been initiated. Even though a lower CDI recurrence rate (4.5%) was achieved with tolevamer than those of metronidazole (23%) and vancomycin (21%), tolevamer did not show promising results in terms of time to resolution of diarrhea and also had a lower rate of clinical success than the therapy with metronidazole and vancomycin^103^. However, anion-binding resins might still be useful as a substitute for antibiotics as an emerging alternative for the treatment of CDI.

## Future perspectives

The current status of CDI management is still worrisome. Available therapeutic agents and effective vaccines for CDI remain limited, and the prevalence of CDI is increasing. Therefore, active prevention will play an increasingly important role in managing this disease more effectively. It has been documented that previous hospitalization, underlying disease, advanced age (≥65 years), and prior use of antibiotics are important risk factors for CDI^1,29^. Based on these risk factors, the Society for Healthcare Epidemiology of America (SHEA) and the Infectious Diseases Society of America (IDSA) have published a series of guidelines for the infection prevention and control of CDI^5^. As most CDIs are hospital-acquired, and *C. difficile* is commonly present in healthcare facilities^1^, measures aiming at preventing the spread of the disease in healthcare facilities should be implemented. Healthcare workers and visitors must use gloves and gowns on entry to a room of a patient with CDI, and they are required to wash hands with soap (or antimicrobial soap) and water after caring for or otherwise having contact with these patients; patients with CDI should be accommodated in a single room with contact precautions, or at least, patients should be cohorted and provided with a dedicated commode for each patient when private rooms are not available^5,24^. Moreover, environmental cleaning and disinfection also are important for reducing *C. difficile* transmission and lowering the incidence of CDI. Any potential environmental sources of *C. difficile* should be determined and removed as far as possible; environmental contamination in areas associated with increased rates of CDI can be addressed by using chlorine-containing cleaning agents or other sporicidal agents; but routine environmental screening for *C. difficile* is not recommended^5^.

Antibiotic stewardship is another important aspect for the prevention and control of CDI, as antibiotic use is regarded as the most important risk factor for CDI^1,29^. Almost all antibiotics have been associated with CDI, but ampicillin, amoxicillin, cephalosporins, clindamycin, and fluoroquinolones are the most frequently cited^1^. Therefore, implementation of an antimicrobial stewardship program is recommended; this program should minimize the frequency and duration of antibiotic therapy in general and specifically restrict the use of those antibiotics most frequently associated with CDI (i.e., cephalosporins and clindamycin) in order to reduce the risk of CDI^5,24^.

The effect of using probiotics to help prevent and control CDI is uncertain at present. While a number of investigators have documented that the use of probiotics composed of various microbial strains had an effect on preventing AAD as well as CDAD^84,85,86,87,88^, other researchers have found no benefit with the administration of probiotics for the prevention of CDI^89,90,91,92,93^. Therefore, the routine use of probiotics for preventing CDI is not recommended at present^1,5^.

In addition to the above recommendations, rapid diagnostic testing of the patients with diarrheal illness acquired in the hospital or associated with antimicrobial therapy is recommended^29^. A hospital-based infection prevention program combined with active surveillance also has been proposed to decrease the incidence of CDI^29^.

It is widely known that *C. difficile* also is able to colonize the intestinal tract of various animals, including dogs, cats, swine, cattle, poultries, and goats^104^. Although there is still a lack of direct evidence that CDI can be transmitted between animals and humans through contact, a large number of studies have demonstrated a close phylogenetic relationship between *C. difficile* originated from humans and animals^44,105,106^. In particular, the hypervirulent 078 isolate has been commonly detected in farm animals as well as in human cases of CDI in North America and Europe. Moreover, this hypervirulent strain also has been frequently detected in meat products^107^. It has been postulated that direct transmission of ribotype 078 strains has occurred between pigs and humans in the Netherlands^108^. Cases of human CDI caused by a livestock-associated ribotype 237 strain recently have been reported in Western Australia^109^. These findings would seem to confirm the possibility that *C. difficile* transmission from animals to humans can occur. Therefore, proper managements of animals, especially those that are in close contact with humans on a daily basis such as household pets, and/or animals that humans consume as food is also important for the prevention of CDI. Measurements such as frequently cleaning of farm animals/pets and their environment, careful handling of animal feces and dead animals, use of antibiotic stewardship in animal husbandry, and required vaccination of both pets and farm animals (when veterinary vaccines become available) are potentially effective and practical methods to prevent zoonotic CDI. In addition, because *C. difficile* and its spores also are commonly found in meats, fish, fruits, and vegetables^104^, good food habits such as washing meats, fish, fruits, and vegetables as well as thoroughly cooking meats and fish before eating may also contribute to the prevention of CDI.

In conclusion, proper and specific therapeutic management of CDI is important for the prevention and control of CDI. A rapid and accurate diagnostic approach for CDI also is a key step for the prevention and control of CDI. Although antimicrobial therapy remains the first choice for CDI, alternate treatment strategies such as FMT and surgical intervention should be considered. Because of the reduction of susceptibility of *C. difficile* to metronidazole, vancomycin, and fidaxocimin, as well as the potential for future problems with resistance, novel antibiotics and therapeutics should continue to be developed. While many guidelines have been suggested for the control and prevention of CDI, proper diagnostic testing and antimicrobial stewardship are the most important factors because antibiotic use remains the most important risk factor for CDI in the absence of effective vaccines. However, some non-medical factors such as proper management of pets and food-producing animals as well as developing good food practices should not be ignored.

## References

[1] Leffler DA, Lamont JT (2015). *Clostridium difficile* infection. N. Engl. J. Med..

[2] Napolitano LM, Edmiston CE (2017). *Clostridium difficile* disease: diagnosis, pathogenesis, and treatment update. Surgery.

[3] Shin, J. H., Chaves-Olarte, E. & Warren, C. A. *Clostridium difficile* infection. *Microbiol. Spectr.* **4**, 10.1128/microbiolspec.EI10-0007-2015 (2016). 10.1128/microbiolspec.EI10-0007-2015 PMC8118380 27337475

[4] Lessa FC, Gould CV, McDonald LC (2012). Current status of *Clostridium difficil*e infection epidemiology. Clin. Infect. Dis..

[5] Cohen SH (2010). Clinical practice guidelines for *Clostridium difficile* infection in adults: 2010 update by the society for healthcare epidemiology of America (SHEA) and the infectious diseases society of America (IDSA). Infect. Control Hosp. Epidemiol..

[6] Bauer MP, Kuijper EJ, van Dissel JT (2009). European Society of Clinical Microbiology and Infectious Diseases (ESCMID): treatment guidance document for *Clostridium difficile* infection (CDI). Clin. Microbiol. Infect..

[7] Debast SB, Bauer MP, Kuijper EJ (2014). European Society of Clinical Microbiology and Infectious Diseases: update of the treatment guidance document for *Clostridium difficil*e infection. Clin. Microbiol. Infect..

[8] Spigaglia P (2016). Recent advances in the understanding of antibiotic resistance in *Clostridium difficile* infection. Ther. Adv. Infect. Dis..

[9] Peng Z (2017). Update on antimicrobial resistance in *Clostridium difficile*: resistance mechanisms and antimicrobial susceptibility testing. J. Clin. Microbiol..

[10] Crobach MJ (2016). European Society of Clinical Microbiology and Infectious Diseases: update of the diagnostic guidance document for *Clostridium difficile* infection. Clin. Microbiol. Infect..

[11] Gerding DN, File TM, McDonald LC (2016). Diagnosis and treatment of *Clostridium difficile* infection. Infect. Dis. Clin. Pract..

[12] Carroll KC, Bartlett JG (2011). Biology of *Clostridium difficile*: implications for epidemiology and diagnosis. Annu. Rev. Microbiol..

[13] Huang B (2014). Real-time cellular analysis coupled with a specimen enrichment accurately detects and quantifies *Clostridium difficile* toxins in stool. J. Clin. Microbiol..

[14] Jin, D. et al. Molecular epidemiology of *Clostridium difficile infection* in hospitalized patients in Eastern China. *J. Clin. Microbiol.* **55**, 801–810 (2017). 10.1128/JCM.01898-16 PMC5328448 27974547

[15] Chow WH (2008). Application of isothermal helicase-dependent amplification with a disposable detection device in a simple sensitive stool test for toxigenic *Clostridium difficile*. J. Mol. Diagn..

[16] Ryder AB (2010). Assessment of *Clostridium difficile* infections by quantitative detection of tcdB toxin by use of a real-time cell analysis system. J. Clin. Microbiol..

[17] Murad, Y. M. et al. False negative results in *Clostridium difficile* testing. *BMC Infect. Dis.* **16**, 430 (2016). 10.1186/s12879-016-1741-6 PMC4992222 27543102

[18] Merz CS (1994). Comparison of four commercially available rapid enzyme immunoassays with cytotoxin assay for detection of *Clostridium difficile* toxin(s) from stool specimens. J. Clin. Microbiol..

[19] Kociolek LK (2017). Strategies for optimizing the diagnostic predictive value of *Clostridium difficile* molecular diagnostics. J. Clin. Microbiol..

[20] Planche T, Wilcox MH (2015). Diagnostic pitfalls in *Clostridium difficile* infection. Infect. Dis. Clin. North Am..

[21] Barbut F (2014). Does a rapid diagnosis of *Clostridium difficile* infection impact on quality of patient management?. Clin. Microbiol. Infect..

[22] Dunwoody, R., Steel, A., Landy, J. & Simmonds, N. *Clostridium difficile* and cystic fibrosis: management strategies and the role of faecal transplantation. *Paediatr. Respir. Rev.* doi:10.1016/j.prrv.2017.03.003 (2017). 10.1016/j.prrv.2017.03.003 28411069

[23] Burnham CA, Carroll KC (2013). Diagnosis of *Clostridium difficile* infection: an ongoing conundrum for clinicians and for clinical laboratories. Clin. Microbiol. Rev..

[24] Surawicz CM (2013). Guidelines for diagnosis, treatment, and prevention of *Clostridium difficile* infections. Am. J. Gastroenterol..

[25] Schroeder LF, Robilotti E, Peterson LR, Banaei N, Dowdy DW (2014). Economic evaluation of laboratory testing strategies for hospital-associated *Clostridium difficile* infection. J. Clin. Microbiol..

[26] Chen S, Gu H, Sun C, Wang H, Wang J (2017). Rapid detection of *Clostridium difficile* toxins and laboratory diagnosis of *Clostridium difficile* infections. Infection.

[27] Gerding DN, Johnson S, Rupnik M, Aktories K (2014). *Clostridium difficile* binary toxin CDT: mechanism, epidemiology, and potential clinical importance. Gut Microbes.

[28] Gilligan PH (2015). Optimizing the laboratory diagnosis of *Clostridium difficile* infection. Clin. Lab. Med..

[29] Smits WK, Lyras D, Lacy DB, Wilcox MH, Kuijper EJ (2016). *Clostridium difficile* infection. Nat. Rev. Dis. Primers.

[30] Martinez-Melendez A (2017). Current knowledge on the laboratory diagnosis of *Clostridium difficile* infection. World J. Gastroenterol..

[31] Deshpande A (2011). Diagnostic accuracy of real-time polymerase chain reaction in detection of *Clostridium difficile* in the stool samples of patients with suspected *Clostridium difficile* Infection: a meta-analysis. Clin. Infect. Dis..

[32] Stockmann C (2015). How well does physician selection of microbiologic tests identify *Clostridium difficile* and other pathogens in paediatric diarrhoea? Insights using multiplex PCR-based detection. Clin. Microbiol. Infect..

[33] Beckmann C, Heininger U, Marti H, Hirsch HH (2014). Gastrointestinal pathogens detected by multiplex nucleic acid amplification testing in stools of pediatric patients and patients returning from the tropics. Infection.

[34] Polage CR (2012). Outcomes in patients tested for *Clostridium difficile* toxins. Diagn. Microbiol. Infect. Dis..

[35] Polage CR (2015). Overdiagnosis of *Clostridium difficile* infection in the molecular test era. JAMA Intern. Med..

[36] Kociolek LK (2017). Impact of a healthcare provider educational intervention on frequency of *Clostridium difficile* polymerase chain reaction testing in children: a segmented regression analysis. J. Pediatr. Infect. Dis. Soc..

[37] Leslie JL, Cohen SH, Solnick JV, Polage CR (2012). Role of fecal *Clostridium difficile* load in discrepancies between toxin tests and PCR: is quantitation the next step in *C. difficile* testing?. Eur. J. Clin. Microbiol. Infect. Dis..

[38] Truong CY (2017). Real-time electronic tracking of diarrheal episodes and laxative therapy enables verification of *Clostridium difficile* clinical testing criteria and reduction of *Clostridium difficile* infection rates. J. Clin. Microbiol..

[39] Senchyna F (2017). *Clostridium difficile* PCR cycle threshold predicts free toxin. J. Clin. Microbiol..

[40] Avila, M. B., Avila, N. P. & Dupont, A. W. Recent advances in the diagnosis and treatment of *Clostridium difficile* infection. *F1000Research* **5**, 10.12688/f1000research.7109.1 (2016). 10.12688/f1000research.7109.1 PMC4755406 26918176

[41] Manzoor SE (2011). Extended multilocus variable-number tandem-repeat analysis of *Clostridium difficile* correlates exactly with ribotyping and enables identification of hospital transmission. J. Clin. Microbiol..

[42] Killgore G (2008). Comparison of seven techniques for typing international epidemic strains of *Clostridium difficile*: restriction endonuclease analysis, pulsed-field gel electrophoresis, PCR-ribotyping, multilocus sequence typing, multilocus variable-number tandem-repeat analysis, amplified fragment length polymorphism, and surface layer protein A gene sequence typing. J. Clin. Microbiol..

[43] Lemee L (2005). Multilocus sequence analysis and comparative evolution of virulence-associated genes and housekeeping genes of *Clostridium difficile*. Microbiology.

[44] Lemee L, Dhalluin A, Pestel-Caron M, Lemeland JF, Pons JL (2004). Multilocus sequence typing analysis of human and animal *Clostridium difficile* isolates of various toxigenic types. J. Clin. Microbiol..

[45] van den Berg RJ, Schaap I, Templeton KE, Klaassen CH, Kuijper EJ (2007). Typing and subtyping of *Clostridium difficile* isolates by using multiple-locus variable-number tandem-repeat analysis. J. Clin. Microbiol..

[46] Franzosa EA (2015). Sequencing and beyond: integrating molecular ‘omics’ for microbial community profiling. Nat. Rev. Microbiol..

[47] Didelot X (2012). Microevolutionary analysis of *Clostridium difficile* genomes to investigate transmission. Genome Biol..

[48] He M (2013). Emergence and global spread of epidemic healthcare-associated *Clostridium difficile*. Nat. Genet..

[49] Eyre DW (2013). Diverse sources of C. difficile infection identified on whole-genome sequencing. N. Engl. J. Med..

[50] Kumar N (2016). Genome-based infection tracking reveals dynamics of *Clostridium difficile* transmission and disease recurrence. Clin. Infect. Dis..

[51] Eyre DW (2017). Comparison of control of *Clostridium difficile* infection in six english hospitals using whole-genome sequencing. Clin. Infect. Dis..

[52] Feher C, Soriano A, Mensa J (2017). A review of experimental and off-label therapies for *Clostridium difficile* infection. Infect. Dis. Ther..

[53] Kali A, Charles MV, Srirangaraj S (2015). Cadazolid: a new hope in the treatment of *Clostridium difficile* infection. Australas. Med. J..

[54] Locher HH (2014). In vitro and in vivo antibacterial evaluation of cadazolid, a new antibiotic for treatment of *Clostridium difficile* infections. Antimicrob. Agents Chemother..

[55] Chilton CH (2014). In vitro activity of cadazolid against clinically relevant *Clostridium difficile* isolates and in an in vitro gut model of C. difficile infection. J. Antimicrob. Chemother..

[56] Gerding DN (2016). Susceptibility of *Clostridium difficile* isolates from a Phase 2 clinical trial of cadazolid and vancomycin in C. difficile infection. J. Antimicrob. Chemother..

[57] Knight-Connoni V, Mascio C, Chesnel L, Silverman J (2016). Discovery and development of surotomycin for the treatment of *Clostridium difficile*. J. Ind. Microbiol. Biotechnol..

[58] Chilton CH (2014). Efficacy of surotomycin in an in vitro gut model of *Clostridium difficile* infection. J. Antimicrob. Chemother..

[59] Lee CH (2016). Surotomycin versus vancomycin for *Clostridium difficile* infection: phase 2, randomized, controlled, double-blind, non-inferiority, multicentre trial. J. Antimicrob. Chemother..

[60] Boix V (2017). Primary outcomes from a phase 3, randomized, double-blind, active-controlled trial of surotomycin in subjects with *Clostridium difficile* infection. Open Forum Infect. Dis..

[61] Leeds JA, Sachdeva M, Mullin S, Dzink-Fox J, Lamarche MJ (2012). Mechanism of action of and mechanism of reduced susceptibility to the novel anti-*Clostridium difficile* compound LFF571. Antimicrob. Agents Chemother..

[62] Sachdeva M, Leeds JA (2015). Subinhibitory concentrations of LFF571 reduce toxin production by *Clostridium difficile*. Antimicrob. Agents Chemother..

[63] Citron DM, Tyrrell KL, Merriam CV, Goldstein EJ (2012). Comparative in vitro activities of LFF571 against *Clostridium difficile* and 630 other intestinal strains of aerobic and anaerobic bacteria. Antimicrob. Agents Chemother..

[64] Trzasko A, Leeds JA, Praestgaard J, Lamarche MJ, McKenney D (2012). Efficacy of LFF571 in a hamster model of *Clostridium difficile* infection. Antimicrob. Agents Chemother..

[65] Ting LS (2012). A first-in-human, randomized, double-blind, placebo-controlled, single- and multiple-ascending oral dose study to assess the safety and tolerability of LFF571 in healthy volunteers. Antimicrob. Agents Chemother..

[66] Mullane K (2015). Multicenter, randomized clinical trial to compare the safety and efficacy of LFF571 and vancomycin for *Clostridium difficile* infections. Antimicrob. Agents Chemother..

[67] Basseres E (2016). Impact on toxin production and cell morphology in *Clostridium difficile* by ridinilazole (SMT19969), a novel treatment for C. difficile infection. J. Antimicrob. Chemother..

[68] Vickers RJ (2016). Ridinilazole: a novel therapy for *Clostridium difficile* infection. Int. J. Antimicrob. Agents.

[69] Goldstein EJ, Citron DM, Tyrrell KL (2014). Comparative in vitro activities of SMT19969, a new antimicrobial agent, against 162 strains from 35 less frequently recovered intestinal Clostridium species: implications for *Clostridium difficile* recurrence. Antimicrob. Agents Chemother..

[70] Vickers RJ (2017). Efficacy and safety of ridinilazole compared with vancomycin for the treatment of *Clostridium difficile* infection: a phase 2, randomised, double-blind, active-controlled, non-inferiority study. Lancet Infect. Dis..

[71] Goldberg EJ (2015). *Clostridium difficile* infection: a brief update on emerging therapies. Am. J. Health Syst. Pharm..

[72] Ofosu A (2016). *Clostridium difficile* infection: a review of current and emerging therapies. Ann. Gastroenterol..

[73] Dieterle MG, Young VB (2017). Reducing recurrence of C. difficile infection. Cell.

[74] Bartlett JG (2017). Bezlotoxumab - a new agent for *Clostridium difficile* infection. N. Engl. J. Med..

[75] Wilcox, M. et al. Bezlotoxumab alone and with actoxumab for prevention of recurrent *Clostridium difficile* infection in patients on standard of care antibiotics: integrated results of 2 phase 3 studies (MODIFY I and MODIFY II). *Open Forum Infect. Dis.* **2**, 67 (2015).

[76] Mathur H, Rea MC, Cotter PD, Ross RP, Hill C (2014). The potential for emerging therapeutic options for *Clostridium difficile* infection. Gut Microbes.

[77] Liubakka A, Vaughn BP (2016). *Clostridium difficile* infection and fecal microbiota transplant. AACN Adv. Crit. Care.

[78] Kassam Z, Lee CH, Yuan Y, Hunt RH (2013). Fecal microbiota transplantation for *Clostridium difficile* infection: systematic review and meta-analysis. Am. J. Gastroenterol..

[79] Youngster I (2014). Oral, capsulized, frozen fecal microbiota transplantation for relapsing *Clostridium difficile* infection. JAMA.

[80] Cohen NA (2016). A retrospective comparison of fecal microbial transplantation methods for recurrent *Clostridium difficile* infection. Isr. Med. Assoc. J..

[81] Orenstein R (2016). Safety and durability of RBX2660 (microbiota suspension) for recurrent *Clostridium difficile* infection: results of the PUNCH CD study. Clin. Infect. Dis..

[82] Martin J, Wilcox M (2016). New and emerging therapies for *Clostridium difficile* infection. Curr. Opin. Infect. Dis..

[83] Gao XW, Mubasher M, Fang CY, Reifer C, Miller LE (2010). Dose-response efficacy of a proprietary probiotic formula of Lactobacillus acidophilus CL1285 and Lactobacillus casei LBC80R for antibiotic-associated diarrhea and *Clostridium difficile*-associated diarrhea prophylaxis in adult patients. Am. J. Gastroenterol..

[84] Maziade PJ, Pereira P, Goldstein EJ (2015). A decade of experience in primary prevention of *Clostridium difficile* infection at a community hospital using the probiotic combination Lactobacillus acidophilus CL1285, Lactobacillus casei LBC80R, and Lactobacillus rhamnosus CLR2 (Bio-K+). Clin. Infect. Dis..

[85] Maziade, P. J., Pereira, P. & Goldstein, E. J. A decade of experience in primary prevention of *Clostridium difficile*infection at a community hospital using the probiotic combination Lactobacillus acidophilus CL1285, Lactobacillus casei LBC80R, and Lactobacillus rhamnosus CLR2 (Bio-K+). *Clin. Infect. Dis*. **60**(Suppl. 2), S144–S147 (2015). 10.1093/cid/civ178 25922400

[86] Johnston BC (2012). Probiotics for the prevention of *Clostridium difficile*-associated diarrhea: a systematic review and meta-analysis. Ann. Intern. Med..

[87] Ratsep M (2017). A combination of the probiotic and prebiotic product can prevent the germination of *Clostridium difficile* spores and infection. Anaerobe.

[88] Wullt M, Hagslatt ML, Odenholt I (2003). Lactobacillus plantarum 299v for the treatment of recurrent *Clostridium difficile*-associated diarrhoea: a double-blind, placebo-controlled trial. Scand. J. Infect. Dis..

[89] Pochapin M (2000). The effect of probiotics on *Clostridium difficile* diarrhea. Am. J. Gastroenterol..

[90] Allen SJ (2013). A high-dose preparation of lactobacilli and bifidobacteria in the prevention of antibiotic-associated and *Clostridium difficile* diarrhoea in older people admitted to hospital: a multicentre, randomised, double-blind, placebo-controlled, parallel arm trial (PLACIDE). Health Technol. Assess..

[91] Allen SJ (2013). Lactobacilli and bifidobacteria in the prevention of antibiotic-associated diarrhoea and *Clostridium difficile* diarrhoea in older inpatients (PLACIDE): a randomised, double-blind, placebo-controlled, multicentre trial. Lancet.

[92] Villano SA, Seiberling M, Tatarowicz W, Monnot-Chase E, Gerding DN (2012). Evaluation of an oral suspension of VP20621, spores of nontoxigenic *Clostridium difficile* strain M3, in healthy subjects. Antimicrob. Agents Chemother..

[93] Nagaro KJ (2013). Nontoxigenic *Clostridium difficile* protects hamsters against challenge with historic and epidemic strains of toxigenic BI/NAP1/027 C. difficile. Antimicrob. Agents Chemother..

[94] Zhang K (2015). The non-toxigenic *Clostridium difficile* CD37 protects mice against infection with a BI/NAP1/027 type of C. difficile strain. Anaerobe.

[95] Gerding DN (2015). Administration of spores of nontoxigenic *Clostridium difficile* strain M3 for prevention of recurrent C. difficile infection: a randomized clinical trial. JAMA.

[96] Ivarsson ME, Leroux JC, Castagner B (2015). Investigational new treatments for *Clostridium difficile* infection. Drug Discov. Today.

[97] Wang Q, Euler CW, Delaune A, Fischetti VA (2015). Using a novel lysin to help control *Clostridium difficile* infections. Antimicrob. Agents Chemother..

[98] Giesemann T, Guttenberg G, Aktories K (2008). Human alpha-defensins inhibit *Clostridium difficile* toxin B. Gastroenterology.

[99] Furci L (2015). New role for human alpha-defensin 5 in the fight against hypervirulent *Clostridium difficile* strains. Infect. Immun..

[100] Bender KO (2015). A small-molecule antivirulence agent for treating *Clostridium difficile* infection. Sci. Transl. Med..

[101] Johnson S (2014). Vancomycin, metronidazole, or tolevamer for *Clostridium difficile* infection: results from two multinational, randomized, controlled trials. Clin. Infect. Dis..

[102] Rodriguez C, Taminiau B, Van Broeck J, Delmee M, Daube G (2016). *Clostridium difficile* in food and animals: a comprehensive review. Adv. Exp. Med. Biol..

[103] Goorhuis A (2008). *Clostridium difficile* PCR ribotype 078: an emerging strain in humans and in pigs?. J. Clin. Microbiol..

[104] Janezic S (2014). International *Clostridium difficile* animal strain collection and large diversity of animal associated strains. BMC Microbiol..

[105] Goorhuis, A. et al *Clostridium difficile* PCR ribotype 078: an emerging strain in humans and in pigs? *J. Clin. Microbiol.* **46**, 1157 (2008). 10.1128/JCM.01536-07 PMC2268365 18326836

[106] Knetsch CW (2014). Whole genome sequencing reveals potential spread of *Clostridium difficile* between humans and farm animals in the Netherlands, 2002 to 2011. Eur. Surveill..

[107] Mc Govern AM (2016). Human *Clostridium difficile* infection caused by a livestock-associated PCR ribotype 237 strain in Western Australia. JMM Case Rep..

[108] Knetsch, C. W. et al. Whole genome sequencing reveals potential spread of *Clostridium difficile* between humans and farm animals in the Netherlands, 2002 to 2011. *Eur. Surveill.* **19**, 20954 (2014). 10.2807/1560-7917.es2014.19.45.20954 PMC4518193 25411691

[109] Mc Govern, A. M. et al. Human *Clostridium difficile* infection caused by a livestock-associated PCR ribotype 237 strain in Western Austr﻿alia.* JMM Case Rep.* **3**, e005062 (2016). 10.1099/jmmcr.0.005062 PMC5330249 28348781

